# Advances in Diagnosis and Treatment of Fetal Alcohol Spectrum Disorders: From Animal Models to Human Studies

**DOI:** 10.35946/arcr.v37.1.07

**Published:** 2015

**Authors:** Nathen J. Murawski, Eileen M. Moore, Jennifer D. Thomas, Edward P. Riley

**Affiliations:** Nathen J. Murawski, Ph.D., is a postdoctoral fellow at the Center for Behavioral Teratology;; Eileen M. Moore, Ph.D., is a research assistant professor in the Department of Psychology;; Jennifer D. Thomas, Ph.D., is associate director at the Center for Behavioral Teratology and a professor in the Department of Psychology;; Edward P. Riley, Ph.D., is director of the Center for Behavioral Teratology and a distinguished professor in the Department of Psychology, all at San Diego State University, San Diego, California.

**Keywords:** Fetal alcohol spectrum disorders, prenatal alcohol exposure, fetal alcohol effects, developmental alcohol exposure, developmental disorder, diagnosis, treatment, intervention, human studies, clinical studies, animal models, literature review

## Abstract

Prenatal alcohol exposure can cause a number of physical, behavioral, cognitive, and neural impairments, collectively known as fetal alcohol spectrum disorders (FASD). This article examines basic research that has been or could be translated into practical applications for the diagnosis or treatment of FASD. Diagnosing FASD continues to be a challenge, but advances are being made at both basic science and clinical levels. These include identification of biomarkers, recognition of subtle facial characteristics of exposure, and examination of the relation between face, brain, and behavior. Basic research also is pointing toward potential new interventions for FASD involving pharmacotherapies, nutritional therapies, and exercise interventions. Although researchers have assessed the majority of these treatments in animal models of FASD, a limited number of recent clinical studies exist. An assessment of this literature suggests that targeted interventions can improve some impairments resulting from developmental alcohol exposure. However, combining interventions may prove more efficacious. Ultimately, advances in basic and clinical sciences may translate to clinical care, improving both diagnosis and treatment.

Alcohol consumption during pregnancy can interfere with both embryonic and fetal development, producing a wide range of outcomes that fall under the rubric of fetal alcohol spectrum disorders (FASD). FASD is the nondiagnostic umbrella term used to refer to the full range of effects that can occur following prenatal alcohol exposure. Such exposure can produce a variety of effects, including physical birth defects, growth retardation, and facial dysmorphism, but the most profound effects are on the developing brain and accompanying cognition and behavior. The disabilities associated with prenatal alcohol are variable, influenced by numerous factors, and can have a life-long impact. Therefore, early diagnosis and intervention are essential for improved clinical outcomes (Streissguth et al. 2004).

Animal models have played a critical role in research on FASD, including studies confirming that alcohol is indeed a teratogen and those providing insights into the mechanisms by which alcohol exerts its teratogenic effect. Researchers have used a wide variety of organisms to model the effects of prenatal alcohol exposure, which mimic both the physical and the behavioral alterations seen in human FASD (Wilson and Cudd 2011). These models allow researchers to experimentally control factors, including alcohol dose, pattern and timing of exposure, nutritional status, maternal factors, and genetics, that are known to influence and contribute to variability in clinical outcomes. Animal models also can help identify better strategies for diagnosing and treating FASD. This review will not directly compare the animal and human data because previous reviews have done this (Schneider et al. 2011). Rather, it will highlight and integrate translational research that might lead to advancements in the diagnosis and treatment of FASD. Furthermore, several psychosocial, academic, and behavioral interventions for FASD that recently have been discussed elsewhere (Paley and O’Connor 2011) are difficult to model in animals and thus will not be reviewed here. Instead, this review focuses on recent pharmacological, nutritional, and exercise interventions that have shown promise in preclinical studies and are progressing toward translation to the clinic.

## Identification and Diagnosis

**Figure 1:**
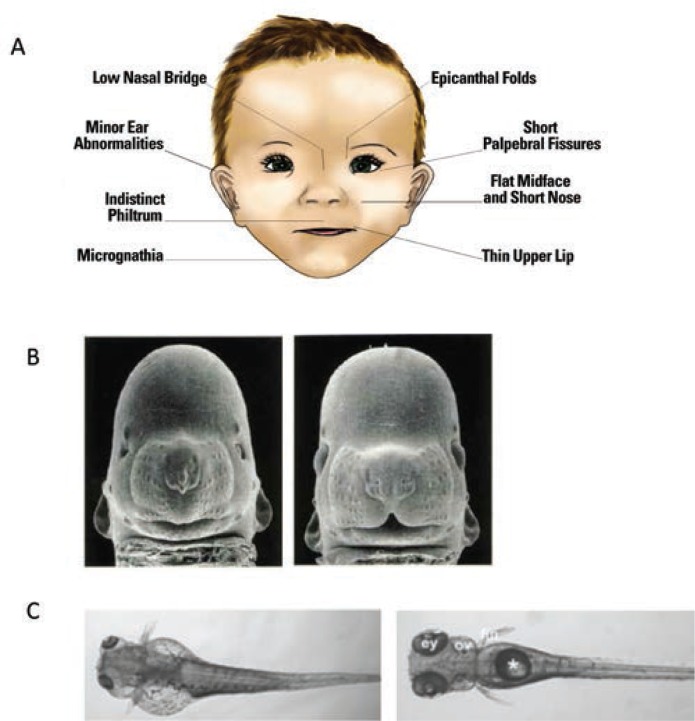
Craniofacial anomalies associated with alcohol exposure during development. **(A)** An illustration of a child with facial features of fetal alcohol syndrome (FAS). **(B)** Left figure shows a mouse with gestational day 7 alcohol exposure: Note small head, small eyes, and lack of a cleft under the nose compared with the control mouse on the right. **(C)** Zebrafish with embryonic alcohol exposure on the left compared with a control on the right. Again notice the small eyes, the smaller head, and the malformed body cavity and fin displacement resulting from alcohol exposure. SOURCE: Figure 1A: Warren et al. 2011. Photos in B are courtesy of Dr. Kathleen Sulik, University of North Carolina at Chapel Hill. Photos in C were taken from Marrs et al. 2010.

To obtain an accurate estimate of FASD prevalence and provide early intervention for affected individuals, it is critical to identify infants prenatally exposed to alcohol. Identification is less problematic on the severe end of the spectrum—where fetal alcohol syndrome (FAS) lies—because it is characterized by obvious growth retardation, central nervous system (CNS) dysfunction, and a specific pattern of craniofacial anomalies (see figure 1A). However, many, if not the majority, of individuals affected by prenatal alcohol exposure do not meet criteria for FAS (Bertrand et al. 2005), yet have significant neurobehavioral impairments (Mattson et al. 2013). These cases are referred to as alcohol-related neurodevelopmental disorders (ARND) and are often difficult to identify because they lack the characteristic facial features and growth retardation seen in FAS. In fact, an ARND diagnosis requires confirmation of prenatal alcohol exposure, which often is unavailable or unreliable (see Riley et al. 2011 for a comparison of various diagnostic schemas for FAS and ARND). Finding novel ways to identify at-risk individuals for disabilities along the spectrum is critical, as is identifying effective interventions to mitigate these cognitive and behavioral effects.

**Figure 2:**
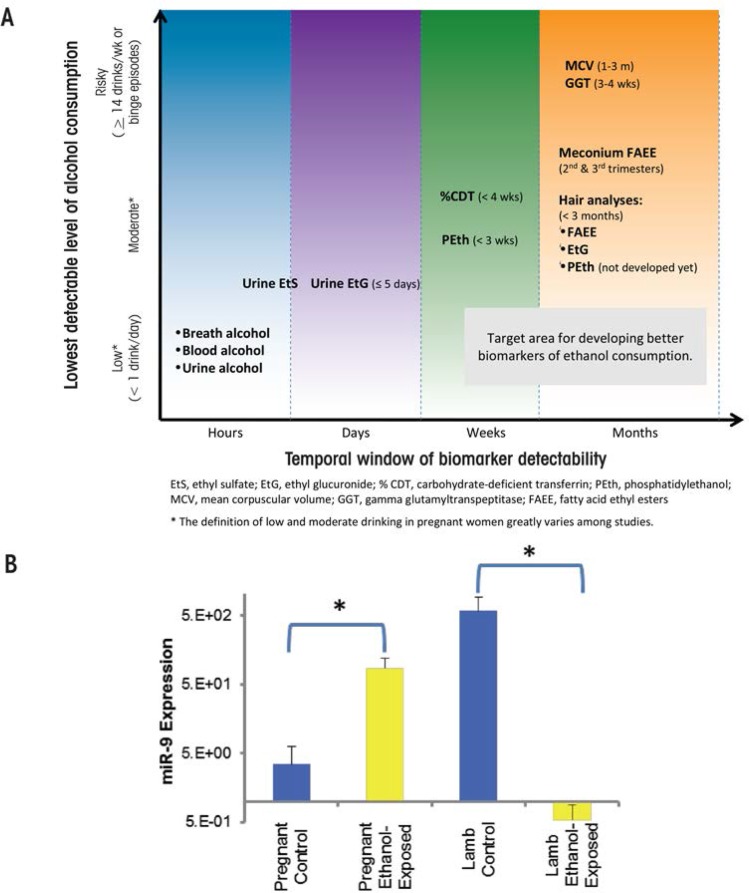
Indirect and direct markers of alcohol exposure. **(A)** Ideally, biomarkers could be both sensitive and specific to alcohol exposure and also indicate the timing and amount of alcohol exposure. This figure shows the period of time, or detection window, during which alcohol consumption can be detected and the lowest levels of alcohol consumption detectable by current alcohol biomarkers. For example, fatty acid ethyl esters are detectable in a variety of biological samples, such as neonatal hair and meconium, for several months after exposure. **(B)** MicroRNAs (miRNAs) may serve as potential biomarkers. Using a sheep model, Dr. Rajesh Miranda has identified several miRNAs that are modified by ethanol. As shown in this panel, miR-9 expression was significantly increased in plasma from the ethanol-exposed pregnant female compared with the control female but significantly decreased in plasma from neonatal lamb compared with controls. Alterations in miR-9 may be indicative of alcohol exposure in the mother, but also may serve as a marker of alcohol-induced injury in the neonate. SOURCE: Figure 2(A): Bakhireva and Savage 2011. Figure 2(B): Modified from Balaraman et al. 2014. NOTE: ^*^ = significantly different from control.

The routine use of objective, validated, and highly specific markers of prenatal alcohol exposure would help improve FASD identification, which currently is hampered by a lack of good information. For example, a recent study (May et al. 2014*a*) found that only 33 percent of the mothers of children given a diagnosis of FAS provided information about their alcohol consumption. In addition, a large number of children with FASD are in adoptive situations or foster care, and there may be little knowledge of their alcohol exposure. Several indirect and direct markers of alcohol exposure (see figure 2A) exist and have been described at length elsewhere (Bakhireva and Savage 2011). Fatty acid ethyl esters, ethyl glucuronide, ethyl sulphate, and the alcohol-derived phospholipid phosphatidylethanol are among several promising metabolic biomarkers. All of these are byproducts of alcohol metabolism, and each is limited by how long after alcohol exposure they are detectable. Another newly identified marker may persist longer than these metabolic markers. As shown in a sheep model, unique circulating microRNAs (miRNA) may help identify individuals consuming alcohol and, importantly, those exposed to alcohol in utero. An initial study suggests that several microRNAs (miRNAs), including miR-9, -15b, -19b, and -20a, are potentially sensitive indices of alcohol exposure in both the pregnant ewe and newborn lamb (Balaraman et al. 2014) (see figure 2B). Researchers are conducting miRNA studies in humans to confirm the sheep findings. If they succeed, miRNAs may provide a new tool to identify alcohol-exposed pregnancies/infants, similar to their use as diagnostic biomarkers in a variety of other disease states (Weiland et al. 2012).

**Figure 3:**
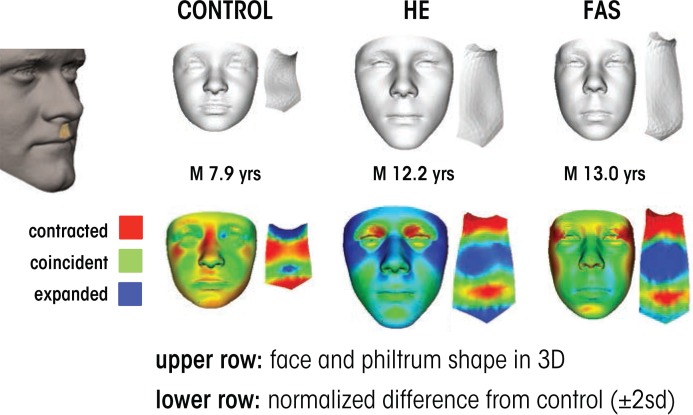
Three-dimensional facial imaging used to detect the effects of prenatal alcohol exposure. Each case shows face and philtrum (ridge under nose) shape as well as heat maps indicating significant regions of difference from age- and sex-matched control subjects. The control case shows an unexposed individual with some flattening across the nasal bridge, a small jaw and a strongly grooved philtrum. The heavily exposed (HE) case is an individual with known exposure without clinically recognized fetal alcohol syndrome (FAS). The overall face size is average or larger and the upper part of philtrum is smooth. The FAS case shows a reduced face size and philtrum smoothness, best revealed in the philtrum heat map; red at outer canthi (outer edge of eye) identifies narrow palpebral fissures.

Other novel FASD diagnostic techniques include ways to identify potential at-risk individuals based upon subtle, subclinical facial features. In particular, researchers have developed a computerized method for detecting facial features using three-dimensional facial imaging and computer-based dense-surface modeling (see figure 3). Hammond and colleagues (Suttie et al. 2013) compared this approach with a standard dysmorphology exam for diagnosing FAS and found a high degree of agreement. The researchers used sophisticated mathematical techniques to characterize the facial features of heavily exposed individuals who did not have facial features that would have led to a diagnosis of FAS using traditional measures. They categorized participants as having facial features that were either “more similar to those with FAS” or “more similar to unexposed controls.” Importantly, the heavily exposed children with FAS-like faces performed at a level similar to the FAS group on neurobehavioral tests, whereas those with more control-like faces exhibited behavioral profiles similar to control subjects. These data were collected on a homogenous ethnic group in South Africa and therefore need to be replicated in other populations. Still, they provide preliminary evidence that this approach may constitute a means to identify at-risk individuals based upon subtle, sub-clinical facial features.

**Figure 4:**
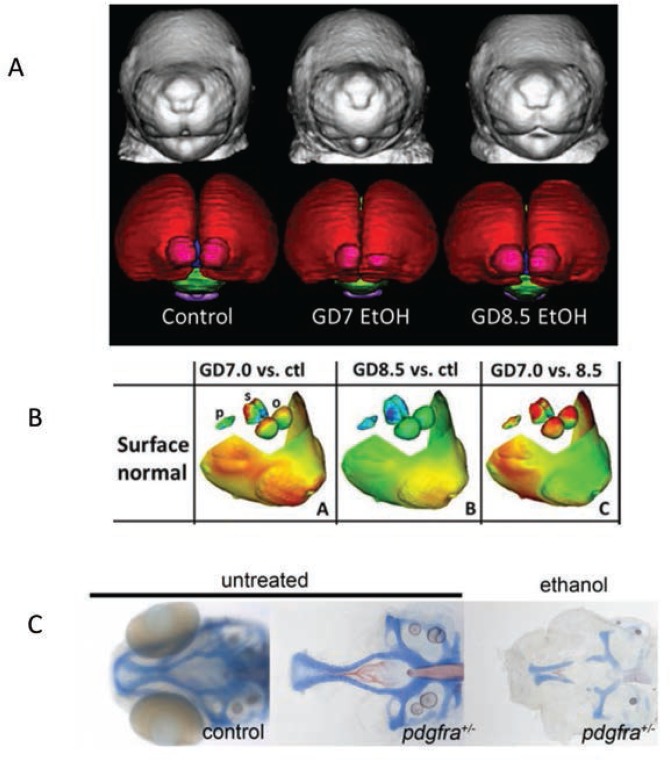
Magnetic resonance imaging (MRI) images showing the differential effect of different timing of exposure on face shape and brain morphology. **(A)** The left panel shows a control, whereas the two other panels show animals exposed on gestation day 7 and gestation day 8.5. The different timing produces differential effects on face and brain. **(B)** An illustration of how the shape analysis shown in figure 3 can be applied to the mouse images. The left panel shows the difference between an animal exposed on gestation day 7 versus a control. Red areas indicate a reduction in size. The middle panel shows gestation day 8 exposure versus control, note the absence of many red areas. The right panel shows the difference between the two exposure times. **(C)** Ethanol interacts synergistically with the *PDGFRA* gene. The two left most figures show an intact embryo and the dissected neurocranium of a stained *PDGFRA* heterozygote displaying normal morphology of the neurocranium. The right most panel shows how ethanol severely disrupts development of the anterior neurocranium and palate of the zebrafish. The homozygote, −/−, (not shown) is even more affected. SOURCE: Photos in A and B are courtesy of Dr. Kathleen Sulik, University of North Carolina at Chapel Hill. Photos in C are courtesy of Dr. Johann Eberhart, University of Texas at Austin.

Developing truly accurate and specific methods for identifying individuals with FASD requires an understanding of the full spectrum of alcohol-related consequences and clarification of the various factors, both protective and permissive, that influence outcome variability. Animal models have provided information on the mechanisms by which alcohol affects facial development and the factors that may make a fetus more susceptible to these facial changes (see figure 1B and C for examples of craniofacial defects in the mouse and zebrafish). In the mouse, for example, alcohol administration on gestational day (GD) 7, equivalent to approximately week 3 postfertilization in a human pregnancy, produces a constellation of facial malformations similar to those seen in FAS. Defects include severe midfacial hypoplasia, shortening of the palpebral fissures, an elongated upper lip, and deficient philtrum (Godin et al. 2010). However, alcohol exposure delayed a day and a half to GD 8.5 produces a distinctly different pattern of malformations, with mild hypoplasia and shortening of the palpebral fissures and upper lip but a preserved philtrum (Lipinski et al. 2012) (see figure 4A and B). These data suggest that maternal alcohol consumption, even before many women are aware that they are pregnant, can cause significant and selective facial alterations in their offspring. The distinctive facial phenotype of FAS depends on the timing of exposure, and other facial characteristics resulting from alcohol exposure during different critical periods are possible.

As with facial dysmorphology, basic science models illustrate that the timing of alcohol administration also produces differing patterns of brain malformations, which again may account for the variability in outcomes. O’Leary-Moore and colleagues (2011) recently reviewed the different brain changes following a single day of alcohol exposure during early fetal development in the mouse using magnetic resonance imaging (MRI). Alcohol exposure on GD 7 was particularly damaging to medial forebrain regions, with relative sparing of mesencephalic and rhombencephalic regions (Godin et al. 2010). The morphological changes induced by alcohol exposure on GD 8 included disproportionate volume reductions in the olfactory bulbs, hippocampus, and cerebellum and relative sparring of the pituitary and septal regions (Parnell et al. 2009). GD 9 exposure produced reductions in cerebellar volume, ventricle enlargement, and shape deviations in the cerebral cortex, hippocampus, and right striatum (Parnell et al. 2013). In contrast, offspring exposed to alcohol on GD 10 displayed enlarged ventricles and disproportionate reductions in cortical volume (O’Leary-Moore et al. 2010). Brain-imaging studies in humans with FASD also find morphological alterations in many of these brain structures (see Moore et al. 2014 for review), which may vary depending on the specific timing of alcohol exposure. These exposure timing–dependent brain changes likely produce different behavioral outcomes, contributing to the variability in impairment seen clinically. Ultimately, understanding the relationship between alcohol exposure parameters and variability in outcome, including different behavioral phenotypes, may improve detection of individuals with FASD.

Recent studies also suggest that the interaction of alcohol with specific genes involved in brain development and the development of facial features may affect the FASD phenotype. A study in zebrafish, for example, examined the interaction of alcohol with the gene for platelet-derived growth factor receptor alpha (Pdgfra) (McCarthy et al. 2013). This gene is involved in cellular migration and proliferation and is necessary for proper migration of neural crest cells, which contribute to the formation of diverse structures, including the face. The researchers found that pdgfra interacts with alcohol to protect against severe craniofacial defects. Specifically, more than 60 percent of zebrafish heterozygous for the pdgfra gene showed cranial facial defects after alcohol exposure compared with only about 10 percent of the alcohol-treated wild-type embryos (figure 4C). A genome-wide genetic scan, using single nucleotide polymorphisms (SNPs), in humans with FASD supports these findings, showing that craniofacial phenotypes seen in FASD are linked to the *PDGFRA* gene (McCarthy et al. 2013). A more recent study in zebrafish found that a gene involved in the development of the embryonic axis, *vangl2,* interacts strongly with alcohol (Swartz et al. 2014). This finding provides another potential gene target to help identify significant sources of variance in terms of susceptibility to the facial characteristics and perhaps changes in brain seen in FASD (see McCarthy and Eberhart 2014 for a recent review of genetic factors involved in FASD).

Basic research in people with FASD also is providing new methods for assessing alcohol’s clinical effects. Studies have identified several relationships between facial measurements and brain structure in FASD (reviewed in Moore et al. 2014). For example, shorter palpebral fissures predict volume reductions in the bilateral ventral diencephalon, a thinner anterior corpus callosum, and a thicker right inferior frontal cortex. The smoothness of the philtrum predicts volumetric reductions in the thalamus and the left pallidum. Facial measures also predict brain maturation patterns: Children with greater facial dysmorphia displayed a linear pattern of cerebral cortex growth, at least from childhood through adolescence, rather than the developmentally appropriate inverted U-shaped trajectory. Continued research examining the relationship between face, brain, and behavioral outcomes resulting from prenatal alcohol eventually may lead to the identification of specific patterns of anomalies that can be used to better identify FASD and improve diagnosis. Moreover, patterns of outcomes may illuminate mechanisms by which alcohol disrupts developmental processes, which can inform treatment strategies. It must be cautioned, however, that the utility of these findings will largely depend on their sensitivity and specificity to alcohol.

## Treatment Strategies

Although no specific treatments exist that are unique for FASD, the similarity between the cognitive and behavioral characteristics of FASD and other disorders provides a framework for treatment development. For example, estimates indicate that anywhere from around 50 percent to over 90 percent of individuals with FASD who have been clinically referred meet diagnostic criteria for attention deficit/hyperactivity disorder (ADHD) (Bhatara et al. 2006; Fryer et al. 2007). One approach would be to treat individuals with FASD with medications, such as stimulants, that have been successful in treating ADHD. However, mixed results have been found with stimulant treatment in clinical studies on FASD. For example, treatment with stimulant medications may reduce hyperactivity, with little evidence for beneficial effects on attention (e.g., Doig et al. 2008). Other studies have noted variable and unpredictable effects (O’Malley and Nanson 2002) or even poorer outcomes (Frankel et al. 2006) in FASD. Animal studies find that perinatal alcohol exposure leads to hyperactivity and that treatment with stimulants later in life increases, rather than attenuates, animals’ spontaneous locomotor behaviors (Hannigan and Berman 2000). Atomoxetine (Strattera), a nonstimulant medication for ADHD, also is often used in the treatment of attention problems in FASD and a clinical trial of its effectiveness in FASD is under way.

Researchers are using their knowledge of the mechanisms underlying alcohol’s toxic effect on the fetus to design preclinical models that test the efficacy of a number of pharmaceutical agents to mitigate alcohol-related impairments (Idrus and Thomas 2011). For example, prenatal alcohol exposure results in deficient activation of cyclic-AMP response element–binding protein (CREB), which can impair brain plasticity, a process of neural change important for brain development, learning, and memory. The pharmaceutical vinpocetine, a vasodilator and anti-inflammatory agent, inhibits the enzyme phosphodiesterase type 1, an action that prolongs CREB activation and thereby strengthens synaptic connections. Studies in animal models find that vinpocetine attenuates alcohol-related impairments in cortical plasticity and reduces learning and memory deficits associated with developmental alcohol exposure (Medina 2011). Clinical trials in humans with dementia have shown some promise and no serious adverse consequences, although results with other disorders, such as ischemic stroke remain inconclusive (Medina 2011). Clinical studies to evaluate this drug in humans with FASD are an important next step.

Preclinical models of FASD also have used neuroprotective peptides to mitigate neuropathologies and behavioral impairments resulting from developmental alcohol exposure. Originally, researchers administered the neuroactive peptides NAP and SAL concurrently with alcohol to pregnant rodents in an attempt to prevent alcohol-induced damage in the offspring. Subsequently, researchers have administered the peptides to adolescent rodents exposed to alcohol prenatally and found that they can reduce deficits in behavioral tasks, such as a T-maze and a Morris water maze (Incerti et al. 2010). The peptides also reversed alcohol-related changes in NMDA receptors in the hippocampus and cortex. These peptides are being developed to treat a number of neurodegenerative diseases and may prove useful in the treatment of FASD.

### Nutritional Interventions

Research clearly shows that nutritional factors influence alcohol’s damaging effects on the fetus. Moreover, it is possible that postnatal nutrition also might influence physical and behavioral outcomes in individuals with FASD.

### Prenatal Nutritional Interventions

Some studies suggest that women who drink during pregnancy have nutritional deficits relative to control subjects. In one study, for example, May and colleagues (2014*b*) examined the nutritional status of a group of South African mothers who gave birth to children with FASD compared with a group of mothers who gave birth to children without FASD. The mothers of children with FASD were more likely to be deficient in several vitamins, including vitamins A, B6, choline, C, D, and E; minerals, including calcium, iron, and zinc; and omega-3 fatty acids. Deficiencies in these micronutrients during pregnancy can contribute to abnormal fetal development (Nyaradi et al. 2013) and may further exacerbate the damaging effects of alcohol on the developing embryo and fetus. In animal models, maternal nutritional deficiencies (e.g., zinc or iron) during pregnancy increase the detrimental effects of prenatal ethanol on brain development and subsequent behavior in offspring. For example, the combined insults of prenatal alcohol exposure and iron deficiency resulted in increased cerebellar apoptosis (cell death), reduced myelin content, and greater impairments in cerebellar-dependent classical eyeblink conditioning compared with either insult alone (Rufer et al. 2012).

Research also finds that nutritional supplementation during pregnancy may attenuate ethanol’s teratogenic effects. In one relatively small study (Avalos et al. 2011), low to moderate alcohol consumption during pregnancy resulted in a twofold increase in small-for-gestational-age infants relative to mothers who abstained. However, the offspring of women who consumed alcohol and reported taking nutritional supplements during pregnancy were no different on these measures than the offspring of abstainers (Avalos et al. 2011). The study reported similar results for preterm births. In a study of pregnant women currently being conducted in the Ukraine, researchers compared the birth outcomes of women given vitamin supplements with those not given supplements. Both groups included women who were consuming alcohol. Although the researchers still are analyzing the results, preliminary reports indicate that the women consuming alcohol and taking micronutrient supplements have a lower rate of babies with FASD than women in the nonsupplement group (Chambers et al. 2013).

Other nutritional interventions target oxidative stress. Alcohol increases oxidative stress, which in turn can initiate a cascade of events that eventually lead to widespread CNS cell loss during development (Brocardo et al. 2011). In rodent models of FASD, pregnant females given nutrients high in antioxidant properties (e.g., vitamin C, vitamin E, omega-3 fatty acids) during the time they also are given alcohol, give birth to offspring with reduced oxidative stress and cell loss, and fewer behavioral impairments (Brocardo et al. 2011; Patten et al. 2013*a*). Although antioxidant treatments in animal models are encouraging, researchers prematurely terminated a clinical trial utilizing high doses of vitamins C and E in women with alcohol-exposed pregnancies because of safety concerns (Goh et al. 2007).

Other studies are examining the role of nutritional supplements on gene transcription. Animal models of FASD demonstrate that prenatal alcohol exposure significantly affects gene transcription through epigenetic modifications (Ungerer et al. 2013). Specifically, alcohol-induced changes in DNA methylation, histone modification, and noncoding RNAs may alter the expression patterns of numerous genes important for neurodevelopment and behavior. Nutrients such as choline, betaine, folic acid, methionine, and zinc can influence these epigenetic profiles and can potentially attenuate alcohol-induced changes to the epigenome. For example, supplemental choline in rats exposed to alcohol during development alters alcohol-related changes in global DNA methylation in the hippocampus and prefrontal cortex (Otero et al. 2012) and significantly attenuates ethanol-induced hypermethylation of genes in the hypothalamus (Bekdash et al. 2013). Additionally, access to a diet supplemented with nutrients that act as methyl donors normalized changes to DNA methylation patterns in embryonic tissue following a single binge exposure to alcohol in early gestation (Downing et al. 2011). These nutrient-induced changes to the epigenome may contribute to the behavioral and cognitive improvements seen in alcohol-exposed rodents following supplementation (see below).

Additional preclinical research indicates that supplementation with beta-carotene (provitamin A), nicotinamide (the amide of vitamin B3), and zinc all may reduce alcohol’s effects on fetal development, including cell loss, fetal dysmorphology, and cognitive impairments (reviewed in Idrus and Thomas 2011). These animal studies highlight the protective effects that nutrient supplementation can have on development during alcohol exposure. Improving the nutritional status of pregnant women, especially those who consume alcohol, will likely result in improved outcomes in offspring.

### Postnatal Nutrient Interventions

**Figure 5:**
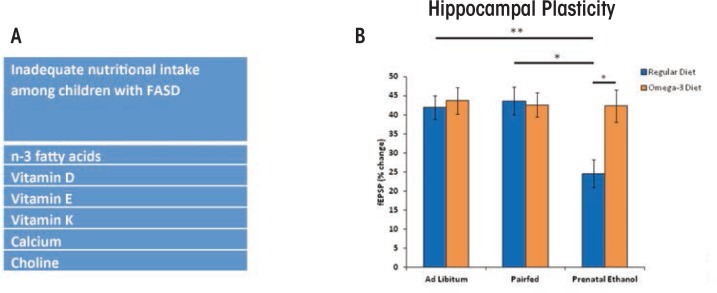
**(A)** Many children with fetal alcohol spectrum disorder (FASD) are not consuming adequate or recommended levels of nutrients (Fuglestad et al. 2013). **(B)** Rodent models have shown that postnatal supplementation with various nutrients, including vitamin D, choline, and omega-3 fatty acids can reduce the severity of FASD. As shown in B, prenatal alcohol exposure in a rodent model impaired hippocampal plasticity, as measured by reduced long-term potentiation (blue bars = normal diet), an effect attenuated with postnatal supplementation with omega-3 fatty acids (orange bars = omega-3 supplemented diet) (Patten, et al. 2013*b*). Such studies illustrate how preclinical and clinical studies may inform one another in the development of effective interventions for FASD. NOTE: ^*^ = significant group differences at *p* ≤ 0.05; ^**^ = significant group differences at *p* ≤ 0.01)

Nutritional status also can affect cognitive development throughout childhood (Bryan et al. 2004). Recent studies have examined the nutritional intake of children with FASD. Based on their dietary habits, many children with FASD are not consuming adequate or daily-recommended amounts of omega-3 fatty acids, vitamin D, and choline (figure 5A) (Fuglestad et al 2013; Werts et al. 2014). Although these studies have some limitations—including low sample sizes, comparison with national data rather than a local control group, and relying on self-reports—they do indicate that individuals with FASD ingest inadequate levels of certain nutrients and therefore may benefit from nutrient supplementation. In rodent models, administering these micronutrients during or shortly following developmental alcohol exposure significantly mitigated ethanol-induced impairments on brain and behavior (figure 5B) (Idrus and Thomas 2011; Patten et al. 2013*b*). For example, animal models have shown that choline can attenuate ethanol’s adverse effects on both brain and behavioral development when administered postnatally, long after alcohol exposure has ceased (Ryan et al. 2008).

Clinical studies currently are underway to examine the effectiveness of choline supplementation in children with FASD. Preliminary results from a study examining choline supplementation in children with FASD aged 2.5–4.9 years suggest that supplemental choline is both feasible and tolerable, with few side effects being reported (Wozniak et al. 2013). The results on behavioral measures should be available soon. In addition to nutrient supplementation, at-risk populations may benefit from better access to food naturally high in nutrients found to improve outcomes in animal studies.

### Exercise Interventions

Exercise has many beneficial effects on brain and behavior outcomes. Reports in both human and rodents indicate that exercise improves learning and memory; increases circulating proteins that support brain function, such as brain-derived neurotrophic factor (BDNF); and, in rodents, increases generation of new neurons in the adult hippocampus (Voss et al. 2013). In addition, clinical studies show beneficial cognitive effects following exercise in normal aging, Alzheimer’s disease, and Parkinson’s disease (reviewed in Yau et al. 2014). No published studies to date have implemented an exercise intervention to improve cognitive and behavioral outcomes in individuals with FASD, but preliminary data and preclinical results are promising, as described below.

Studies suggest that running may enhance learning and memory in rodents prenatally exposed to alcohol. Rodents will run multiple kilometers per day when they have access to a running wheel, making it ideal for an exercise intervention. Indeed, access to a running wheel significantly attenuates spatial learning and memory impairments in adult rats exposed to alcohol during development (Christie et al. 2005; Thomas et al. 2008). In addition, these improvements in cognitive function following exercise are associated with exercise-induced enhancements in BDNF and adult hippocampal neurogenesis, both of which are influenced by developmental alcohol exposure (Gil-Mohapel et al. 2010).

However, the long-term effects of short periods of exercise may be limited. For example, increases in BDNF return to normal levels within 2 weeks following exercise (Gil-Mohapel et al. 2010). That said, the benefits of exercise may be prolonged through additional environmental experiences, such as those provided by raising animals in an enriched, stimulating environment. In fact, Hamilton and colleagues (2014) have found that the combination of wheel running followed by enrichment significantly increases adult neurogenesis relative to wheel running alone in alcohol-exposed rats. Similarly, exercise plus enrichment mitigates alcohol-induced impairments on behavioral tasks, such as trace eyeblink conditioning and contextual fear conditioning. Behavioral improvement was associated with increases in adult neurogenesis (Hamilton et al. 2014). In addition, specific motor training can have beneficial effects on the structure and function of the cerebellum among rodents exposed to alcohol prenatally (Klintsova et al. 2000).

In translating these preclinical findings to human studies, researchers may need to tailor their exercise interventions to accommodate some of the motor impairments evident in FASD. A recent meta-analysis of motor skills in children and adolescents with FASD reported impairments in balance, motor coordination, and ball skills (Lucas et al. 2014).

A number of clinical research programs are using these findings to develop motor training and/or exercise interventions and investigate their efficacy in individuals with FASD. None have published results yet, except in abstract form. The following are two promising examples:

- Researchers at the University of Washington are using sensorimotor training via a virtual-reality system to try to improve motor deficits. Participants stand on a moveable surface, wearing virtual-reality goggles as the program attempts to train them to use sensory information for balance (Jirkowic et al. 2014).
- Researchers at the University of the Fraser Valley are using strength-based interventions in an attempt to improve motor skills and cognitive function in FASD. In this intervention, clinicians create a physical activity and motor skills program based on an individual child’s strengths, with the hope that such training may generalize to some aspects of executive functioning, attention, and visuospatial processing in children with FASD (Keiver et al. 2014).

## Conclusion

FASD can be difficult to treat for a number of reasons. First, identifying individuals with prenatal alcohol exposure can be a challenge. Although the characteristics of FAS are well defined, alcohol-affected children who do not meet the criteria for FAS or for whom exposure histories are unknown are more difficult to ascertain. Children who are diagnosed earlier have improved clinical outcomes (Streissguth et al. 2004), highlighting the need for early identification. Although there are methodological and ethical concerns that must be addressed, sensitive and specific biomarkers of exposure or effect would improve identification. Continued research examining the interrelations among alcohol-induced face and brain malformations and neurocognitive outcomes using both human and animal models may yield novel means for identification and/or novel specific targets for interventions.

Overall, studies with animal models of FASD demonstrate a wide array of benefits of pharmacological, nutritional, and environmental interventions to both brain structure/function and behavior. However, relatively few clinical studies have evaluated such treatments in FASD. There are some important potential limitations to these treatments. First, many of the treatments have very specific targets and consequences, whereas the range of deficits in FASD is quite varied. For example, in animal models of FASD, nutritional supplementation with choline has a greater positive effect on hippocampal function compared with cerebellar function; in contrast, motor training may be better able to target cerebellar effects in this population. Interventions that use multiple intervention strategies (e.g., nutrition and exercise) as well as more traditional interventions (educational, speech, occupational and/or physical therapies) may mitigate a wider range of cognitive impairments when translated to clinical cases of FASD. Given the numerous successes in identifying potential interventions in preclinical research, the upcoming years should increase translation of these findings to clinical research and eventually to health care settings.

## Notes

Glossary

Apoptosis: A process of programmed cell death.
Brain-derived neurotrophic factor (BDNF): A protein secreted in the brain to support the survival of neurons; it plays a role in the growth, differentiation, and maintenance of these cells.
Cerebellum: An area of the brain important for coordinating motor function, as well as playing a role in simple learning and attention.
Corpus callosum: A wide bundle of fibers that connects the left and right hemispheres of the brain.
Cortex: The outer layer of the brain that is composed of folded gray matter and associated with perception, voluntary movement, and integration of information to support cognitive functions such as memory, language, and abstract thinking, among others.
cAMP response element–binding protein (CREB): A protein that binds to certain stretches of DNA and influences activation of genes.
Epigenetics: The study of factors that affect gene expression without directly changing the DNA.
Epigenome: Chemical changes to the DNA and histone proteins that affect gene expression.
Ethyl glucuronide: A byproduct of alcohol metabolism formed in the body after alcohol consumption.
Ethyl sulphate: A byproduct of alcohol metabolism formed in the body after alcohol consumption.
Fatty acid ethyl esters: The products of a reaction between ethanol and fatty acid cells.
NMDA receptors: A receptor in the brain activated by the neurotransmitter glutamate. Among its many roles, NMDA receptors help control synaptic plasticity (the ability of the brain to change and evolve), learning and memory.
Oxidative stress: When there is an imbalance between the body’s production of reactive oxygen species (free radicals), and antioxidants, which defend against reactive oxygen species.
Pallidum: Refers to the globus pallidus, a subcortical brain structure involved in the regulation of voluntary movement.
Palpebral fissures: The opening between the upper and lower eyelids; length is measured as the distance between the inner to outer eye corners.
Peptide: Chains of 10 to 50 amino acids.
Philtrum: The typically vertical groove between the upper lip and nose.
Phosphatidylethanol: A metabolite of alcohol, created when phospholipase D interacts with alcohol.
Teratogen: A substance that interferes with development and causes birth defects.
Thalamus: A part of the vertebrate brain made up of two symmetrical halves deep in the middle of the brain. Among other roles, it is involved in relaying sensory and motor signals to the cerebral cortex, and regulating consciousness, sleep, and alertness.
